# Human Papilloma Virus vaccine and cervical cancer screening acceptability among adults in Quebec, Canada

**DOI:** 10.1186/1471-2458-7-304

**Published:** 2007-10-25

**Authors:** Chantal Sauvageau, Bernard Duval, Vladimir Gilca, France Lavoie, Manale Ouakki

**Affiliations:** 1 Quebec Regional Public health department, Health and Social Services Agency, Quebec, Canada; 2 Quebec Institute of Public Health, Quebec, Canada; 3 Public health research center, Laval University Hospital Center, Quebec, Canada; 4 Social and Preventive Medicine Department, Laval University, Quebec, Canada

## Abstract

**Background:**

The Pap test has been used for cervical cancer screening for more than four decades. A human papillomavirus (HPV) vaccine has been approved for use in Canada and is commercially available now. These two preventive interventions should be considered simultaneously. General population support is an important factor for the successful combination of these interventions. The study had two objectives: 1) To assess practices, beliefs, and attitudes regarding Pap test screening and HPV immunization; 2) To identify socio-demographic factors for Pap screening and vaccine acceptability.

**Methods:**

In 2006, 500 adults were invited to participate in a telephone survey in the region of Quebec City (urban and rural population, 600 000), Canada. Some neutral and standardized information on Pap test and HPV was provided before soliciting opinions.

**Results:**

471 adults (18–69 year-olds) answered the questionnaire, the mean age was 45 years, 67% were female, and 65% had college or university degree. Eighty-six percent of women had undergone at least one Pap-test in their life, 55% in the last year, and 15% from 1 to 3 years ago. Among screened women, the test had been performed in the last three years in 100% of 18–30 year-olds, but only in 67% of 60–69 year-olds (P < 0.0001). Only 15% of respondents had heard of HPV. Eighty-seven percent agreed that HPV vaccines could prevent cervical cancer, 73% that the vaccine has to be administered before the onset of sexual activity, 89% would recommend vaccination to their daughters and nieces. Among respondents < 25 years, 91% would agree to receive the vaccine if it is publicly funded, but only 72% would agree to pay $100/dose.

**Conclusion:**

There is an important heterogeneity in cervical cancer screening frequency and coverage. Despite low awareness of HPV infection, the majority of respondents would recommend or are ready to receive the HPV vaccine, but the cost could prevent its acceptability.

## Background

Genital Human papillomavirus (HPV) infection is the most common sexually transmitted infection (STI). More than 50% of sexually active women have been infected with genital HPV at some time in their life [1-5]. Infection prevalence of up to 82% has been reported in adolescent and young adult women[4]. Approximately 15 virus genotypes cause virtually all cases of cervical cancer [6-8].

Cervical cancer screening and treatment have been in place for more than four decades. There is now a new approach: vaccination against two of the most common disease-causing HPV genotypes. The HPV vaccines, if widely used, have the potential to prevent thousands of cases of cervical cancer worldwide, as well as to substantially reduce costs and emotional stress associated with abnormal screening test (Pap) results[9,10]. The vaccines target adolescent girls and young women. Several surveys have been done with target groups as adolescents and parents [11-16]. The surveys show that awareness about HPV is very low and that many women are shocked to discover that cervical cancer is caused by a STI.

In previously surveyed populations, estimated vaccine acceptability was high [11-16]. However, the vaccine up-take by adolescents and young women may be influenced by opinions of other family members and friends.

In preparation for the implementation of HPV vaccine in Canada, we measured acceptability, attitudes, beliefs and practices related to cervical cancer screening and vaccination, in Canadian adults.

We hypothesize that regular cancer screening, a positive attitude toward vaccines in general and the HPV vaccine in particular, as well as perceived personal risk for cervical cancer and seriousness of its consequences would be associated with a higher HPV immunization acceptance and vaccine up-take.

## Methods

This HPV survey was conducted as a part of a study on a number of personal health-related beliefs, attitudes and practices, including smoking, nutrition, exercise, cardiac disease and cervical cancer prevention.

### Study population

Survey population included adult females and males aged 18–69 years. The potential participants were recruited during February and March 2006 in 11 private outpatient clinics in the region of Quebec City. At the moment of the survey, 84 (of the 500 in the region) family physicians were practicing in these clinics. Most Canadian family physicians (78%) practice at least part of their time in private outpatient clinics[17]. Two specially trained research assistants recruited potential participants in the clinic's waiting rooms. If the individual agreed to participate in the planned telephone survey, their personal contact information was collected and a written consent form was signed. During the following two weeks, a polling firm contacted participants for a telephone interview. The Survey required 13 to 15 minutes to complete.

### Questionnaire

The questionnaire was built using elements of the Health Belief Model [18]. It was pre-tested on 10 outpatients and adjusted before the study began. Out of 56 survey items, 16 were relevant to this paper: 3 items on Pap screening, 3 on cervical cancer, 1 on HPV infection, 1 on vaccination in general, and 8 on eventual HPV vaccination. It took 4–6 minutes to fill out the cervical cancer screening – HPV part of the questionnaire. Because of the hypothesis of low awareness regarding Pap screening and HPV infection, some short, neutral and standardized information was given to participants before asking related questions. For Pap test it was "A Papanicolaou test (Pap test) is an examination of the cervix; the physician takes a sample of cells with a little stick or a brush and sends it to a laboratory to screen for cervical cancer". For HPV it was "The HPV is a sexually transmitted infection and a necessary cause of cervical cancer".

### Statistical analysis

Proportions were compared by using chi-square or Fisher exact test. Trends were evaluated with Cochran-Armitage test. All tests of significance were two-tailed and significance was set at 5% level. SAS Institute software (version 9.1) was used for data statistical analysis.

The Research Ethics Board Committee of Laval University Hospital Centre approved the research protocol.

## Results

Eleven out of 12 solicited clinics agreed to participate in the study and 685 potential participants were initially recruited (536 in 9 urban and 149 in 2 rural areas). Eight individuals refused to respond to the questionnaire and 27 telephone numbers were invalid. As predetermined by the study protocol, no further telephone calls were made when 500 interviews were completed. Among those interviewed, 471 (94.2%) were age eligible and their answers were included in the analysis. The participants' average age was 44.8 years and 67% were female. Sixty five percent of participants had college (32%) or university (33%) degree.

### Women's opinions on cervical cancer

Fifty-seven percent of women were afraid of developing cervical cancer sometime in their life, and 93% thought cervical cancer has serious consequences. Cervical cancer related anxiety and perceived seriousness did not vary by age group or level of schooling.

### Cervical cancer screening

Eighty-six percent of participating women had had at least one Pap test during their life (Table 1). Among all female respondents, 55% had their last Pap test during the previous year, 15% from one to three years ago, and 16% three or more years ago. The cumulative coverage with at least one Pap test was lower in women less than 30 years (68%) compared to older age groups (90%–93%) (P < 0.0001). However, when an analysis of screening in the three previous years was done among those who had at least one Pap test, a significant decrease (p < 0.0001) in screening up-take with increasing age was observed: 100%, 86%, 74% and 67%, respectively in those under 30, 30–44, 45–59, and 60–69 years. The two main opportunities to have a Pap test were the annual check-up (79%) and gynaecological problems (10%).

**Table 1:** Pap test coverage

Had a test	18–29 years (N = 77)	30–44 years (N = 86)	45–59 years (N = 102)	60–69 years (N = 52)	Total (N = 317)
At some time in their life	68 (65–78)	90 (81–95)	93 (86–97)	92 (81–98)	86 (81–89)
<1 year ago	58 (47–70)	62 (51–72)	53 (43–63)	40 (27–55)	55 (49–60)
1 to 3 years ago	9 (4–18)	15 (8–24)	16 (9–24)	21 (11–35)	15 (11–19)
>3 years ago	0 (0–5)	13 (7–22)	25 (17–34)	31 (12–45)	16 (13–21)

Percentages are presented

### Population HPV awareness

Only 15% of respondents had heard about HPV before the interview. The proportion increased from 7% in those with lower than college level of schooling, to 14% and 24% in those with college and university schooling level, respectively (P for trend = 0.0006). No significant differences by sex (P = 0.7) or age group (P = 0.1) were observed on this issue. However, the proportion of women who had heard about HPV was significantly higher among those with at least one Pap test (17%) than those with none (4%) (P = 0.02).

### Attitudes and beliefs about vaccines in general and the HPV vaccine in particular

Ninety percent of respondents agreed with the statement that vaccines in general are effective against infections, but only 47% strongly agreed. A significantly higher proportion (63%) of 60–69 year-olds strongly agreed with vaccine usefulness compared to 34% of 18–29 year-olds (P < 0.0001) (Table 2). Results for the HPV vaccine were in the same range: 87% agreed with the statement that the vaccine would reduce the risk for cervical cancer (Table 3). No significant difference between genders was observed regarding attitudes and beliefs about vaccines in general (P = 1.0) or the HPV vaccine (P = 0.6). Most young women (83%) under 30 years were ready to accept HPV vaccination. However, the need to pay $100 per dose diminished the acceptability of the HPV vaccine in the youngest group (18 to 25 years). The proportion of those who strongly agreed to accept the vaccine fell from 56% to 28% (Table 4). Seventy-three percent of respondents agreed with the statement that the HPV vaccine should be administered to preadolescents before the onset of sexual activity, and 86% would recommend the vaccination to their daughters and/or nieces. No differences between genders were observed on these issues (P = 0.4 and 1.0, respectively). However, the proportion of those who would recommend the vaccine to their daughters and/or nieces was related to the level of schooling and the time since the last Pap test: 90%, 84%, and 83% by those with less than college level of schooling, college and university degree, respectively (P for trend = 0.045); 82%, 85%, and 96% by those who had a Pap test < 1 year, ; 1 to 3 years; and > 3 years ago, respectively (P for trend = 0.03).

**Table 2:** Perceived vaccine usefulness in protection against infectious diseases

	N	Strongly Agree	Somewhat Agree	Somewhat Disagree	Strongly Disagree
Age group (years)					
18–29	104	34 (25–43)	53 (43–62)	13 (6–19)	1 (0–3)
30–44	118	42 (33–50)	48 (39–57)	8 (3–14)	2 (0–4)
45–59	154	48 (40–56)	41 (33–49)	10 (5–14)	1 (0–3)
60–69	91	63 (53–73)	32 (22–41)	4 (1–11)	1 (0–3)
Schooling					
< College	162	56 (49–64)	35 (28–43)	7 (3–11)	1 (0–3)
College	148	36 (29–44)	51 (43–59)	11 (6–16)	1 (0–3)
University	157	45 (37–52)	45 (37–53)	9 (5–14)	1 (0–3)

Percentages and 95% CI are presented

**Table 3:** Perceived HPV vaccine usefulness against cervical cancer

	N	Strongly Agree	Somewhat Agree	Somewhat Disagree	Strongly Disagree
Age group (years)					
18–29	103	39 (29–48)	44 (34–53)	15 (8–21)	3 (0–8)
30–44	117	43 (34–52)	43 (34–52)	11 (5–17)	3 (0–7)
45–59	152	55 (47–63)	35 (27–42)	9 (5–14)	1 (0–3)
60–69	88	59 (49–69)	31 (21–40)	10 (4–17)	0 (0–4)
Schooling					
< College	162	62 (55–70)	28 (21–35)	8 (4–12)	1 (0–3)
College	148	41 (33–49)	40 (32–48)	15 (9–21)	4 (1–7)
University	150	42 (34–50)	47 (39–55)	11 (6–16)	1 (0–2)

Percentages and 95% CI are presented

**Table 4:** HPV vaccine acceptability by young women

Taking for granted efficacy and safety of HPV vaccines: I would agree to receive the vaccine if	N	Strongly Agree	Somewhat Agree	Somewhat Disagree	Strongly Disagree
Free of charge					
18–25 years	43	56 (41–71)	35 (21–49)	7 (0–15)	2 (0–7)
26–30 years	34	41 (25–58)	32 (17–48)	15 (3–27)	12 (1–22)
Have to pay 100$ per dose					
18–25 years	39	28 (14–42)	44 (28–59)	23 (10–36)	5 (0–12)
26–30 years	25	28 (10–46)	56 (37–75)	16 (2–30)	0 (0–13)
If recommended by physician					
18–25 years	43	79 (67–91)	16 (5–27)	5 (0–11)	0 (0–14)
26–30 years	34	50 (33–67)	32 (17–48)	12 (1–23)	6 (0–14)

Percentages and 95% CI are presented

Nineteen percent of respondents under 30 years versus 47% among 60–69 years-old (P < 0.001) perceived HPV vaccination as an incentive for earlier onset of sexual activity (Table 5). Ninety percent of women and 88% of men (P = 0.5) would agree with vaccination of men if it protects women against cervical cancer.

**Table 5:** Perceived HPV vaccine use and consequences

HPV vaccine	Strongly Agree	Somewhat Agree	Somewhat Disagree	Strongly Disagree
Should be given to preadolescents before the onset of sexual activity	39 (35–44)	33 (29–37)	20 (16–24)	7 (5–10)
Would recommend the vaccine to their daughters/nieces	48 (44–53)	37 (32–41)	11 (8–14)	3 (1.8–5)
Should be given to females and males	57 (52–61)	32 (28–37)	9 (6–11)	2 (1–3)
Would encourage earlier sexual debut	12 (9–15)	19 (15–22)	40 (36–45)	29 (25–33)

Percentages and 95% CI are presented; 471 respondents

## Discussion

Despite low awareness of HPV infection, our findings suggest that most young women would accept a vaccine that protects against cervical cancer, especially if it is free of charge and recommended by a physician. The need to pay $100 per dose decreases by half young women's vaccine acceptance. These results show that a potential risk for inequitable preventive care may emerge if HPV vaccines are by individual patient purchase. The Canadian National Advisory Committee on Immunization[19] recommends the use of the HPV vaccine in young females between the ages of 9 and 26. Therefore, the need to pay for the vaccine might substantially decrease the HPV vaccine up-take.

The proportion of respondents who have heard about HPV (15%) has not changed from what was reported in Ontario adolescents (13%)[20]. These findings are also in the same range with those reported in other countries [21-23]. However, in our study, a higher level of schooling was associated with more awareness about HPV.

Overall, opinions on vaccine usefulness in the prevention of infectious diseases are positive. However, as in a recent USA survey [21-23], a disturbing trend of less positive attitudes toward vaccination in younger and more educated populations was noted.

Certain results present a challenge concerning the HPV vaccine use and its potential consequences. Willingness to recommend the vaccine to daughters/nieces was similar to that obtained in UK parents (80%)[23], and was higher when compared to US parents' willingness (55%–67%) [24]. However, in our study, only 72% of respondents agreed that the vaccine should be administered to preadolescents, and 31% expressed concerns about possible earlier onset of sexual activity if the vaccines were administered early in life. These results suggest that HPV vaccine up-take by preadolescents should not be taken for granted. Contrary to our initial hypothesis, a higher proportion of females who had less regular screening tests were willing to recommend an HPV vaccine to their daughters/nieces. Future educational messages and awareness of HPV will play a key role in vaccine acceptance.

Limitations of this study include the non-random sample of participants recruited in outpatient clinics, the absence of knowledge on non participant characteristics and the limited recruitment area. Since HPV vaccines will be administered in a primary care setting, we concur with the other investigators[25], that it is useful to use clinic-based samples. Due to the exploratory nature of the present study, we did not ask questions about previous Pap test results, parenthood or sexual behaviour. Although we used Health Belief Model approaches in the questionnaire construction, we did not collect sufficient information for eventual behaviour predictions. More elaborate studies are needed for this purpose.

The large sample size, high rate of participation, recruitment in both urban and rural areas, and results obtained both in women and men make this survey results useful for planning public health interventions regarding cervical cancer screening and eventual HPV vaccine implementation.

Larger periodic nationwide studies on acceptability, knowledge and attitudes about prevention issues will be needed.

## Competing interests

The author(s) declare that they have no competing interests.

## Authors' contributions

CS conceived the study, coordinated the data collection and helped to draft the manuscript. BD participated in the design of the study and helped to draft the manuscript. VG participated in the design of the study and wrote the first draft of the manuscript. FL participated in the design of the study and helped to draft the manuscript. MO performed the statistical analysis and helped to draft the manuscript. All authors read and approved the final manuscript.

## Pre-publication history

The pre-publication history for this paper can be accessed here:



## References

[B1] Ho GY, Bierman R, Beardsley L, Chang CJ, Burk RD (1998). Natural history of cervicovaginal papillomavirus infection in young women. N Engl J Med.

[B2] Kotloff KL, Wasserman SS, Russ K, Shapiro S, Daniel R, Brown W, Frost A, Tabara SO, Shah K (1998). Detection of genital human papillomavirus and associated cytological abnormalities among college women. Sex Transm Dis.

[B3] Franco E, Harper DM (2005). Vaccination against human papillomavirus infection: A new paradigm in cervical cancer control. Vaccine.

[B4] Brown DR, Shew ML, Qadadri B, Neptune N, Vargas M, Tu W, Juliar BE, Breen TE, Fortenberry JD (2005). A longitudinal study of genital human papillomavirus infection in a cohort of closely followed adolescent women. J Infect Dis.

[B5] Franco EL, Villa LL, Sobrinho JP, Prado JM, Rousseau MC, Desy M, Rohan TE (1999). Epidemiology of acquisition and clearance of cervical human papillomavirus infection in women from a high-risk area for cervical cancer. J Infect Dis.

[B6] Stanley M HPV and pathogenesis of cervical cancer. Eur J Obstet Gynecol Reprod Biol.

[B7] Giordano G, D'Adda T, Gnetti L, Froio E, Merisio C, Melpignano M (2006). Detection of human papillomavirus in organs of upper genital tract in women with cervical cancer. Int J Gynecol Cancer.

[B8] Schiffman M, Castle PE (2003). Human papillomavirus: epidemiology and public health. Arch Pathol Lab Med.

[B9] Harper DM (2004). Why am I scared of HPV?. CA Cancer J Clin.

[B10] Colombo N The wider impact on women's health more than cervical cancer?. Eur J Obstet Gynecol Reprod Biol.

[B11] Constantine NA, Jerman P (2007). Acceptance of human papillomavirus vaccination among Californian parents of daughters: a representative statewide analysis. J Adolesc Health.

[B12] Waller J, Marlow LA, Wardle J (2006). Mothers' attitudes towards preventing cervical cancer through human papillomavirus vaccination: a qualitative study. Cancer Epidemiol Biomarkers Prev.

[B13] Dempsey AF, Zimet GD, Davis RL, Koutsky L (2006). Factors that are associated with parental acceptance of human papillomavirus vaccines: a randomized intervention study of written information about HPV. Pediatrics.

[B14] Lazcano-Ponce E, Rivera L, Arillo-Santillan E, Salmeron J, Hernandez-Avila M, Munoz N (2001). Acceptability of a human papillomavirus (HPV) trial vaccine among mothers of adolescents in Cuernavaca, Mexico. Arch Med Res.

[B15] Zimet GD, Mays RM, Winston Y, Kee R, Dickes J, Su L (2000). Acceptability of Human Papillomavirus Immunization. J of Women's health & Gender-based medicine.

[B16] Zimet GD, Blythe MJ, Fortenberry JD (2000). Vaccine characteristics and acceptability of HIV immunization among adolescents. Int J STD AIDS.

[B17] Savard I, Rodrigue J (2001). La pratique professionnelle des médecins de famille au Québec et au Canada. Le Médecin du Québec.

[B18] Rosenstock IM (1974). Historical origins of the health belief model. Health Education Monographs.

[B19] Dobson S, Deeks S, Money D (2007). Déclaration sur le vaccin contre le virus du papillome humain. CCDR RMTC.

[B20] Dell DL, Chen H, Ahmad F, Stewart DE (2000). Knowledge about human papillomavirus among adolescents. Obstet Gynecol.

[B21] Denny-Smith T, Bairan A, Page MC (2006). A survey of female nursing students' knowledge, health beliefs, perceptions of risk, and risk behaviors regarding human papillomavirus and cervical cancer. J Am Acad Nurse Pract.

[B22] Moreira ED, Oliveira BG, Ferraz FM, Costa S, Costa Filho JO, Karic G (2006). Knowledge and attitudes about human papillomavirus, Pap smears, and cervical cancer among young women in Brazil: implications for health education and prevention. Int J Gynecol Cancer.

[B23] Brabin L, Roberts SA, Farzaneh F, Kitchener HC (2006). Future acceptance of adolescent human papillomavirus vaccination: a survey of parental attitudes. Vaccine.

[B24] Davis K, Dickman E, Ferris D, Dias J (2004). Human papillomavirus vaccine acceptability among parents of 10 to 15 year old adolescents. Journal of lower genital tract disease.

[B25] Zimet GD, Perkins SM, Sturm LA, Bair RM, Juliar BE, Mays RM (2005). Predictors of STI vaccine acceptability among parents and their adolescent children. J Adolesc Health.

